# Relative Visual Oscillation Can Facilitate Visually Induced Self-Motion Perception

**DOI:** 10.1177/2041669516661903

**Published:** 2016-08-05

**Authors:** Shinji Nakamura, Stephen Palmisano, Juno Kim

**Affiliations:** Inter-Departmental Education Center, Nihon Fukushi University, Mihama, Japan; School of Psychology, University of Wollongong, New South Wales, Australia; School of Optometry and Vision Science, University of New South Wales, Sydney, Australia

**Keywords:** vection, self-motion perception, relative motion, oscillation advantage

## Abstract

Adding simulated viewpoint jitter or oscillation to displays enhances visually induced illusions of self-motion (vection). The cause of this enhancement is yet to be fully understood. Here, we conducted psychophysical experiments to investigate the effects of different types of simulated oscillation on vertical vection. Observers viewed horizontally oscillating and nonoscillating optic flow fields simulating downward self-motion through an aperture. The aperture was visually simulated to be nearer to the observer and was stationary or oscillating in-phase or counter-phase to the direction of background horizontal oscillations of optic flow. Results showed that vection strength was modulated by the oscillation of the aperture relative to the background optic flow. Vertical vection strength increased as the relative oscillatory horizontal motion between the flow and the aperture increased. However, such increases in vection were only generated when the added oscillations were orthogonal to the principal direction of the optic flow pattern, and not when they occurred in the same direction. The oscillation effects observed in this investigation could not be explained by motion adaptation or different (motion parallax based) effects on depth perception. Instead, these results suggest that the oscillation advantage for vection depends on relative visual motion.

## Introduction

Optic flow is a visual stimulus generated by the motion of an observer relative to other objects in the environment. The projection of optic flow on the retina is capable of inducing the illusory experience of self-motion in completely stationary observers, known as vection (Brandt, Dichgans & Koenig, 1973; see also Palmisano, Allison, Schira, & Barry, 2015 for alternative definitions of this term). Researchers have used vection measurement as a powerful tool to examine the perceptual mechanisms responsible for the experience of physical self-motion. Although self-motion perception is multisensory in nature, vection demonstrates that visual information alone is sufficient to generate the experience of self-motion without other nonvisual self-motion senses (see Riecke, 2010 and Palmisano et al., 2015 for reviews).

Early studies assumed that vection should be facilitated by minimizing any visual-vestibular sensory conflicts about the physical nature of self-motion (Brandt, Dichgans, & Büchele, 1974; Melcher & Henn, 1981; Wong & Frost, 1981; Young, Dichgans, Murphy, & Brandt, 1973; Zacharias & Young, 1981). According to this view, visual simulations of constant velocity self-motion should induce optimal vection because the vestibular system is specialized for detecting self-accelerations and cannot distinguish between constant linear self-motion and remaining stationarity (Howard, 1986). Although vection appears to be strengthened under many conditions where the expected visual-vestibular conflicts are thought to be reduced (e.g., Melcher & Henn, 1981; Young et al., 1973), Palmisano and coworkers have repeatedly shown that vection is increased (not reduced) by adding simulated self-acceleration to constant velocity displays (see Palmisano, Allison, Kim, & Bonato, 2011 for a review). In the earliest of these studies, Palmisano, Gillam, and Blackburn (2000) found that the vection in depth induced by radially expanding optic flow was strengthened by the addition of visually simulated horizontal or vertical viewpoint jitter (similar to the effects of camera shake). This jitter advantage for vection has been confirmed under a variety of different stimulus settings (see Palmisano et al., 2011 for a review of this research).

The jitter advantage for vection has been found to be robust to changes in the amplitude, frequency, and direction of the jitter. Adding horizontal, vertical, or even diagonal simulated viewpoint jitter has very similar effects on the vection in depth induced by radial flow (e.g.,Palmisano, Allison, & Pekin, 2008; Palmisano et al., 2000). This jitter advantage for vection also does not appear to be due to cognitive factors, such as participant expectations or instructional demands (see Palmisano & Chan, 2004). Whereas the early research was conducted using visually simulated viewpoint jitter (random, broadband simulated head perturbations), later research showed that vection could be improved in a similar fashion by adding visually simulated viewpoint oscillation (periodic simulated head perturbations) to the radial optic flow (i.e., the oscillation advantage; e.g., Nakamura, 2010; Palmisano et al., 2008). Over the years, these robust jitter and oscillation advantages for vection have generated interest because their study should improve understanding of the mechanisms underlying visual-vestibular interactions during self-motion perception (e.g., Snowden, 2000).

Despite their robustness, the perceptual mechanisms underlying these jitter or oscillation advantages are still not fully understood. It is therefore necessary to examine the relationship between jitter or oscillation and other factors which have been hypothesized to be important for visually mediated self-motion perception. Accordingly, Nakamura (2012) analyzed the role that stimulus size and eccentricity plays in the jitter advantage for vection. It is well known that the size and eccentricity of the visual stimulus are among the most critical factors in determining vection (e.g., Brandt et al., 1973), and hence, the analysis of these effects in relation to the jitter advantage should also be important. The results of Nakamura’s (2012) psychophysical experiments indicated that jitter enhancement is maximal in conditions where the observer’s central visual field is stimulated by the jittering motion.^1^ It was originally believed that peripheral visual motion induces stronger vection (e.g., Brandt et al., 1973; Delmore & Martin, 1986). However, more recent studies suggest that vection is independent of the stimulus eccentricity (e.g., Nakamura, 2001; Nakamura & Shimojo, 1998; Post, 1988). It is also widely accepted that larger areas of visual motion stimulation induce stronger vection (e.g., Nakamura & Shimojo, 1998). However, Nakamura (2012) found that the jitter advantage for vection has rather different dependencies in terms of stimulus size, compared with standard nonjittering vection.

Relative motion is one potential property of optic flow that appears to be important for the onset and strength of vection. Vection is often stronger when a static visual reference is presented along with the moving pattern (which results in relative motion between them) compared with conditions where the moving pattern is presented alone (i.e., no object relative motion; e.g., Howard & Howard, 1994; Nakamura, 2006a; Nakamura & Shimojo, 1999). Similarly, it has been reported that the strength and direction of vection are determined as a function of the relative motion between the moving pattern and the static visual target (Howard & Heckman, 1989).

This study investigated the effects of relative motion on the oscillation advantage for vection. We examined the following stimulus situation: a moving visual background pattern viewed through a visually simulated nearby aperture. While the background pattern always moved vertically (consistent with constant downwards vection), the aperture and the background pattern could also be set to oscillate independently of each other (thereby increasing the amount of relative oscillation). If oscillating (and also jittering) vection shares the same dependency on relative visual motion as standard nonoscillating vection, then the oscillation advantage should be: (a) stronger in conditions where the visual stimulus contains strong relative oscillation among its components and (b) weaker when there is less relative visual oscillation. We conducted four related experiments to tease apart the contributions of relative oscillatory motion in either the perpendicular direction (Experiment 1), or parallel (Experiment 2), to the principal (vertical) direction of simulated self-motion. We also investigated the potential roles of visual motion adaptation (Experiment 3) and perceived depth (Experiment 4) on the vection effects observed in Experiments 1 and 2.

## Experiment 1

Experiment 1 tested whether viewpoint oscillation advantages can be altered or modified by the relative oscillation of a moving aperture. In addition to a centrally placed stationary fixation point, we introduced a visually-simulated viewing aperture, through which participants were able to observe a globally coherent optic flow pattern. The stimulus pattern was a random-dot pattern which moved upward at a constant speed while also oscillating horizontally in some conditions. The position of the viewing aperture could also be oscillated horizontally either together with, or independently of, the pattern’s oscillation. This experimental setup enabled us to systematically manipulate the relative oscillation between the aperture and the stimulus pattern. If the relative oscillation plays a role in visual self-motion perception, then we predict that the aperture’s oscillation in front of the oscillating random-dot pattern should modulate or alter the strength of the induced vection.^2^

Two recent studies have shown that vection can be enhanced by providing a fixation target that oscillates independently of the vection inducer (e.g., Nakamura, 2013a; Palmisano, Kim, & Freeman, 2012). Even though the optic flow itself did not contain any acceleration, the observer’s pursuit eye movements in this situation appear to have added global oscillation to the retinal flow, which enhanced vection in a similar fashion to adding an oscillating optic flow component to the vection inducer. In both of these studies, the fixation target not only had a role in terms of controlling eye movements but also served as a reference for relative motion against the flow pattern. In the present study, a visual component other than the fixation spot, namely the simulated aperture, was set to oscillate in front of the visual flow pattern. As the fixation spot was always statically located at in the center of the screen, this allowed us to determine the effects of relative motion on vection independently of the observer’s eye movements (and their flow-on consequences in terms of the retinal image motion).

### Materials and Method

#### Participants

Eighteen undergraduate volunteers (4 males and 14 females with ages ranging from 17 to 52 years old) at the University of Wollongong participated in the experiment. All participants had normal or corrected-to-normal visual acuity and reported no deficits in either their visual or vestibular perception. None of the participants had previous experience participating in vection experiments and all were naïve as to the purpose of this experiment. The University of Wollongong Ethics Committee approved the study in advance, and each subject provided written informed consent before participating in the study.

#### Stimulus and apparatus

**Figure 1.**
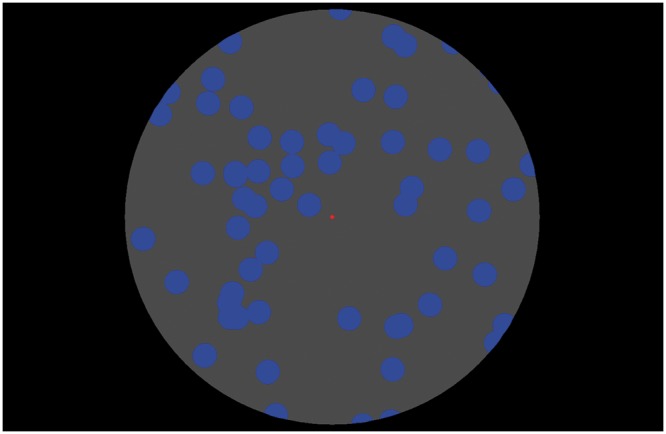
Snapshot of the visual stimulus employed in the experiments.

**Figure 2.**
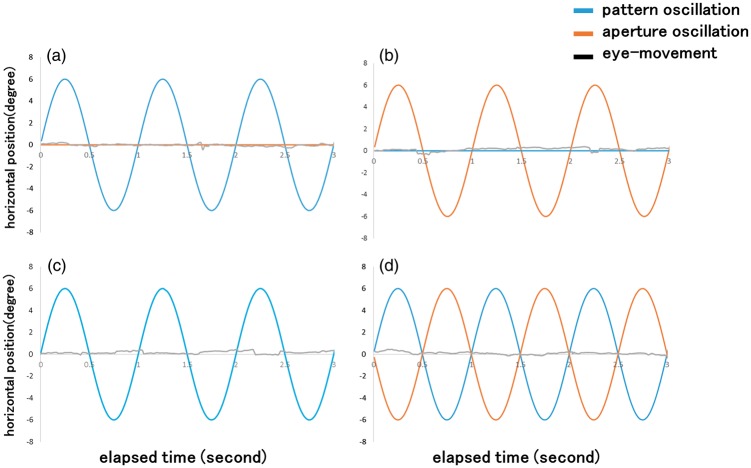
Samples of eye-movement data. Horizontal observer eye movements were recorded via infrared video oculography (Oculog; Kim, Schiemer, & Narushima, 2007) with a sampling rate of 60 Hz. (a) and (b) indicate samples of eye-movement records (gray line) in conditions where only the pattern or the aperture was set to oscillate. (c) and (d) represent the in-phase and the counter-phase aperture oscillation conditions where both the pattern and aperture were oscillated. In each of these stimulus conditions, the observer was able to keep their eyes static (thanks to the fixation spot), despite of the presence of the oscillatory motions of the pattern and the aperture.

The visual stimulus employed in the experiment was composed of a visually simulated (near) aperture and (far) random-dot pattern. The random-dot pattern consisted of randomly positioned blue dots (4.6 cd/cm^2^) which moved upward at a constant speed of 30°/s, and (in some conditions) also oscillated horizontally in a sinusoidal fashion with an amplitude of 6° and a frequency of 1 Hz. The radii of the dots contained in the pattern varied randomly across the display (ranging from 0.5° to 1.0° in visual angle). The background of the random-dot pattern was black (0.5 cd/cm^2^). The aperture, through which the participants observed the motion of the random-dot pattern, was created by superimposing a peripheral annular area of uniform dark gray texture onto the display (2.6 cd/cm^2^). The result was that the random-dot pattern was only visible within a central circular area of the display which had a radius of 30°. Figure 1 shows a snapshot of the visual stimulus employed in this experiment. In some conditions, the horizontal position of the aperture was oscillated around the center of the screen with an identical amplitude and frequency as the random-dot pattern; however, the phase of the oscillation was also manipulated. A 0.5° red fixation spot (luminance: 6.4 cd/m^2^) was presented at the center of the screen. Participants were requested to always fixate their eyes on this spot throughout the experimental trial. Eye-movement recordings of eight participants confirmed that the presence of this fixation spot was sufficient to exclude potential artefacts of unintended eye movements during experimental trials (Figure 2). Demo movies of the visual stimuli employed in the experiment are also provided as supplementary material. The random-dot pattern was always perceived to be located behind the aperture—confirmed in the debriefing sessions (also confirmed in Experiment 4 where observers reported perceived depth of the visual stimulus, instead of perceived self-motion).

These visual stimuli were generated and controlled by a personal computer equipped with an OpenGL compatible graphics card and presented on a 2 × 2 m screen using a DLP projector (Mitsubishi XD400U) with a resolution of 1024 × 768 and a refresh rate of 60 Hz. All experiments were conducted in a darkened experimental room; the visual stimulus display was the sole light source in the room. The participants sat on a chair in front of the screen with a viewing distance of 150 cm. They observed the stimulus with their eyes fixed to a physical (real) viewing booth, so that they could not see the edges of the screen (although all of the visual display, including black background, was still visible through the booth), and their heads were roughly held stationary because the viewing booth was fixed to an immovable frame.

There were two independent variables in this experiment. The first was the pattern oscillation. In the *pattern oscillation condition*, the upward moving random-dot pattern also oscillated horizontally. In the *no pattern oscillation condition*, the random-dot pattern only moved upward. The other independent variable was the aperture oscillation, and there were three different conditions. In the *in-phase aperture oscillation condition*, the aperture was set to oscillate horizontally with the same amplitude, frequency and phase as the random-dot pattern, and thus, the aperture oscillation was completely synchronized with that of the random-dot pattern. In the *counter-phase aperture oscillation condition*, the phase of the aperture’s oscillation was shifted 180° with respect to the random-dot pattern. Thus, in this condition, the random-dot pattern and the aperture were oscillated in opposite directions. There was also a *no aperture oscillation condition*, in which the aperture never moved and remained static in the central position.

There were six different stimulus conditions in total (2 [pattern oscillation, no pattern oscillation] × 3 [in-phase, counter-phase, and no aperture oscillations]). It should be noted that in the no pattern oscillation condition, the in-phase and counter-phase aperture oscillations both resulted in identical visual stimulation. In these conditions, the random-dot pattern moved upward without horizontal oscillation and the aperture oscillated horizontally in front of it. Both of these conditions generate identical patterns of retinal motion.

#### Procedure

The participant’s task was to report perceived self-motion (vection) by pressing a mouse button. It was emphasized that he or she needed to hold the button down as long as their vection experience continued and release it if the vection dropped out during the trial. Directly after each 30-second stimulus observation, participants were also required to rate the strength of the vection experienced during that trial using method of magnitude estimation (Stevens, 1957). Participants were first exposed to the standard stimulus, which was identical to displays used in conditions with no aperture and no pattern oscillation. The vection experienced with this standard stimulus was assigned a strength of “50” (the modulus), and participants were instructed to estimate the strength of vection in subsequent experimental trials based on this reference. For example, if participants perceived that the vection strength for a trial was 30% stronger than that experienced for the standard condition, they evaluated it as “65.” In experimental trials where the participants did not experience any vection, estimates were assigned as “0.” To establish the modulus for these strength estimates and allow the participants to become familiar with the experimental procedure, a single observation of the standard stimulus was executed before the experimental trials. Each participant was then presented with three repeats of the six different experimental conditions in a randomized order. They could request to take a short rest and observe the standard stimulus whenever they were required.

Vection onset latency was calculated based on the time it took from the start of the trial until the observer first pressed a mouse button. Because there were very few vection dropouts, total vection durations were highly correlated with vection onset latencies (Pearson’s coefficient of correlation was −0.75 in the case of the current experiment). Accordingly, we only analyzed vection onset latencies and vection strength ratings (not total vection durations) in order to avoid redundancy. Prior to conducting these analyses, vection onset latencies and vection strength ratings were averaged across observers. Then a two-way repeated measurement analysis of variance (rANOVA) with a factorial design of 2 (pattern oscillation) × 3 (aperture oscillation) was then applied for each of the vection indices.

### Results and Discussion

**Figure 3.**
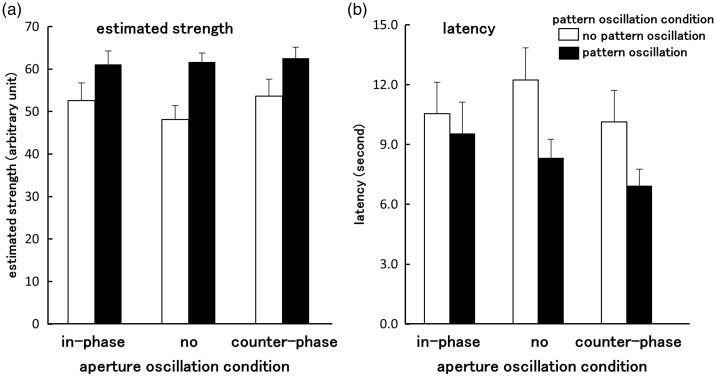
Average estimated strength (a) and latency (b) of vection measured under each stimulus condition (Experiment 1). Error bars indicate *SEMs*. The open bars indicate the no pattern oscillation conditions in which the random-dot pattern was set to move purely upward at a constant speed. The solid bars indicate the pattern oscillation conditions in which the pattern moved upward with horizontal oscillation. The abscissa indicates the oscillations of the aperture. In the in-phase oscillation condition, the aperture oscillated with the same phase as the random-dot pattern. In the counter-phase oscillation condition, the phase of the aperture’s oscillation was shifted 180° with respect to the random-dot pattern. In the no aperture oscillation condition, the aperture remained static in the central position.

Figure 3 shows averaged latency and estimated strength of vection measured in each experimental condition. rANOVAs revealed a significant main effect of the pattern oscillation for both vection indices (latency: *F*(1, 17) = 10.67, *p = *.005, pη^2 ^= .39; estimation: *F*(1, 17) = 19.43, *p* < .001, pη^2 ^= .53). Latency was shorter, and estimated strength was higher in the pattern oscillation conditions than in the no oscillation condition. Thus, it can be concluded that vertical vection was stronger in the presence of horizontal random-dot pattern oscillation than without it. This replicates the findings of previous studies that presented optic flow without an aperture (e.g., Nakamura, 2010; Palmisano et al., 2008). rANOVAs also indicated a significant main effect of aperture oscillation for latency (*F*(2, 34) = 3.45, *p* = .043, pη^2 ^= .17) but not for estimated strength (*F*(2, 34) = 2.30, *p* = .12, pη^2 ^= .12). Latency was shorter in the aperture oscillation conditions than in the no aperture oscillation conditions.

A significant two-way interaction between pattern oscillation and aperture oscillation was also found for latency (*F*(2, 34) = 3.56, *p* = .039, pη^2 ^= .17). However, this interaction only approached marginal significance level for estimated strength (*F*(2, 34) = 2.76, *p* = .077, pη^2 ^= .14). Due to the significant interaction observed for latency, a further analysis of variance was conducted in order to examine the simple main effects of aperture oscillation separately for each pattern oscillation condition. Simple main effects were significant in the no pattern oscillation condition (*F*(2, 34) = 4.58, *p* = .017, pη^2 ^= .21), and marginally significant in the pattern oscillation condition (*F*(2, 34) = 3.00, *p* = .063, pη^2 ^= .15). Multiple comparisons using Bonferroni correction (*α* = 0.05) were executed, and the main results of these comparisons are discussed later.

In the no pattern oscillation condition, vection onset latency was significantly shorter for the aperture oscillation conditions (in-phase and counter-phase) than for the no aperture oscillation condition. This indicates that horizontal aperture oscillation was able to reduce vection onset latencies even when the background random-dot pattern moved purely upward (i.e., when the pattern had no horizontal oscillation of its own and only the aperture motion provided any visual acceleration). In the pattern oscillation condition, latency was significantly shorter in the counter-phase aperture oscillation than in the in-phase condition. Adding pattern oscillation in no aperture oscillation and counter-phase aperture oscillation conditions also significantly reduced vection latencies (relative to no pattern oscillation conditions). However, adding pattern oscillation provided no significant benefit when this pattern oscillation was in-phase with the aperture oscillation.

The above findings all appear consistent with the hypothesis that relative motion is critical for the oscillation advantage in vection. Relative oscillation in the counter-phase condition was twice that of the no aperture oscillation condition; by contrast, no relative oscillation was visible between the aperture and the pattern in the in-phase condition. Thus, the pattern oscillation might have improved vection because it increased the level of relative oscillation between the aperture and pattern in conditions when the aperture was stationary or when the aperture was moving in a counter-phase fashion. However, there was less benefit of adding pattern oscillation when it was in-phase with the aperture oscillation, as the aperture and pattern were seen to move together as one (i.e., there was less relative motion between these two visual features than during aperture oscillation alone; the only relative motion would have been between these visual features and the static fixation point). When there was no aperture oscillation, vection latency was shorter in the pattern oscillation condition than in the no pattern oscillation condition. Relative oscillation between the aperture and the random-dot pattern only occurred in the former condition, not in the latter one. Thus, the results in the no aperture condition were also consistent with our relative motion hypothesis.

It should be noted that the simple main effect of the aperture oscillation was only marginally significant when there was pattern oscillation, which could be attributed to residual pattern motion relative to fixation. Although there was no relative oscillation between the aperture and the random-dot pattern in the in-phase aperture oscillation condition, pattern oscillation may have significantly increased vection (compared with the standard nonoscillating condition) because of the its motion relative to the static fixation spot (the possible effect of the fixation spot as a reference for relative motion will be discussed further in section General Discussion). It is possible that additional effects of the relative aperture oscillation might have been somewhat obscured due to the presence of this fixation spot increasing the strength of the baseline vection.

The results of Experiment 1 were discussed earlier in relation to the relative oscillation between the random-dot pattern and the aperture (where the aperture provided a spatial frame of reference for interpreting all of the available visual motion). However, it was also possible that the motion of the aperture might have independently affected the observer’s perception of self-motion. To address this possibility, we conducted an additional experiment with nine naïve observers who did not take part in Experiment 1. In this experiment, the random-dot pattern was always static and only the aperture was oscillated horizontally. Stimulus dimensions and the procedure used to measure perceived self-motion were both identical to those of the main experiment. Although vection was consistently induced by the standard stimulus, none of the nine observers reported experiencing vection with this new stimulus. Thus, it can be concluded that the effects of aperture oscillation found in Experiment 1 were due to the relative oscillation between the random-dot pattern and the aperture, not the aperture oscillation by itself. The presence of a static random-dot pattern presented behind the oscillating near aperture is known to strongly inhibit vection (as it was shown to do here). Research has shown that a moving foreground cannot induce self-motion perception in the presence of a static background (e.g., Nakamura & Shimojo, 1999).

The results of this experiment revealed that, even with the eyes held stationary, the relative oscillation between the two visual stimuli had the potential to facilitate self-motion perception (at least in terms of the vection time course; the reason why similar effects could not be confirmed for estimated strength will be discussed later in section General Discussion).

## Experiment 2

In this second experiment, the effects of relative oscillation on vection were investigated further using conditions where the stimulus pattern moved upward at a constant speed and oscillated *vertically* behind an aperture which could also be set to oscillate *vertically*. Hence, the oscillation occurred in the same direction as the constant velocity pattern motion (unlike Experiment 1 where any oscillation always occurred in an orthogonal direction to the constant velocity pattern motion). Previous studies suggest that jitter or oscillation advantages for vection only arise when the additional acceleration is orthogonal to the main (constant speed) motion of the visual inducer; these advantages either disappeared or were substantially diminished when the added acceleration was parallel to the main motion (e.g., Nakamura, 2010; Palmisano et al., 2008).

### Methods

#### Participants

Ten naïve undergraduate volunteers who did not participate in Experiment 1 acted as observers in Experiment 2 (5 men and 5 women, aged 18–46 years old).

#### Stimuli

The stimuli and apparatus employed in Experiment 2 were almost identical to those in Experiment 1. The sole difference was the direction of the oscillation assigned to the random-dot pattern and the aperture. In Experiment 2, the random-dot pattern was set to move upward at a constant speed (30°/s) while oscillating vertically (frequency: 1 Hz, amplitude: 6°). The aperture was also set to oscillate vertically in some conditions.

#### Procedure

The procedures used to measure vection strength and latency in Experiment 2 were exactly the same as those used in Experiment 1. There were two independent variables as in Experiment 1: pattern oscillation and aperture type. Trials for each stimulus condition were repeated 3 times in a randomized order, and thus, each participant executed 18 trials in total. The modulus for vection strength estimation was set using the same visual stimulus used in Experiment 1 (no aperture or pattern oscillation). An estimate of 50 was assigned for vection strength as strong as self-motion perception induced by the standard stimulus.

### Results and Discussion

**Figure 4.**
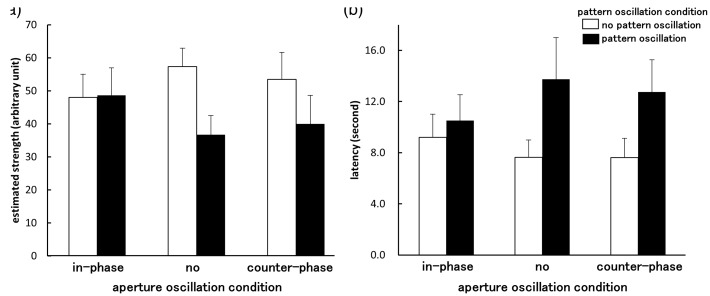
Average estimates of vection strength (a) and latency (b) measured under each stimulus condition (Experiment 2). Error bars indicate *SEMs*.

Figure 4 shows the averaged latency and estimated strength of vection for each of the stimulus conditions. rANOVA revealed that only the main effects for pattern oscillation were significant (latency: *F*(1, 9) = 5.23, *p* = .048, pη^2 ^= .37; estimation: *F*(1, 9) = 9.15, *p* = .014, pη^2 ^= .50). Vection was found to be weaker (as indicated by longer latencies and lower estimates) in the vertical pattern oscillation condition than in the no pattern oscillation condition. Thus it appears that vertical vection can be impaired by adding vertical oscillation to the constant vertical pattern motion. In previous research, adding jitter or oscillation along the same axis as the main constant motion component did not significantly alter vection strength (there was no facilitation or suppression of vection—see Nakamura, 2010; Palmisano et al., 2008). However, in the current experiment, adding vertical pattern oscillation significantly decreased the strength and increased the latency of vertical vection. These effects might have been due to the fact that, in the experiments reported in this article, the random-dot pattern (the primary inducer of vection) was always presented centrally and had a circular area of limited size. As a result, the vection induced might have been somewhat weaker compared with previous studies with large or full field presentations. It is well known that vection strength increases as a function of stimulus size (e.g., Nakamura & Shimojo, 1998). Thus, the relatively weak vection observed for the control condition (constant visual motion without the pattern oscillation) might have been responsible for vection decline when vertical pattern oscillation was added.

The main effects of the aperture oscillation were not significant for either vection measure (latency: *F*(2, 18) = .15, *p* = .86, pη^2 ^= .017; estimation: *F*(2, 18) = .14, *p* = .87, pη^2 ^= .015). The interactions between pattern oscillation and aperture oscillation also did not reach significance (latency: *F*(2, 18) = 1.90, *p* = .18, pη^2 ^= .17; estimation: *F*(2, 18) = 2.10, *p* = .15, pη^2 ^= .18). These results suggest that vection was unaffected by aperture oscillation and combined aperture-and-pattern oscillation when the aperture oscillation occurred parallel to the direction of perceived self-motion.

## Experiment 3

In Experiment 3, we measured durations of the motion aftereffects generated by each of the stimuli used previously in Experiment 1. The rationale for conducting this experiment was that a number of vection studies have argued that jitter and oscillation might alter vection by affecting the observer’s adaptation to the visual motion stimulation (e.g., Kim & Khuu, 2014; Palmisano et al., 2000, 2012; Seno, Palmisano, & Ito, 2011). This is because a complicated moving stimulus, which contains visual acceleration (i.e., jitter or oscillation) and transiently varies its local velocity, should be difficult for a local motion detector to adapt to (compared with a simple moving pattern that has a constant speed). Thus, if the visual oscillations assigned to either the random-dot pattern or the aperture altered vection in Experiment 1 by reducing adaptation to the visual inducer, then evidence of this should be seen in terms of the durations of the motion aftereffects observed directly after exposure to the optic flow.

### Methods

#### Participants

Eight observers participated in this experiment (4 men and 4 women, aged 18–46 years old). One of these was an author (S. N.). The other seven observers were naïve undergraduate volunteers who did not participate in either Experiments 1 or 2.

#### Stimuli

The stimuli and apparatus employed in Experiment 3 were identical to those in Experiment 1—with the following exceptions. The visual stimulus was set to move for 30 seconds (adaptation phase), then all motion stopped and a static version of the visual stimulus was continuously presented on the screen (test phase).

#### Procedure

Participants kept the mouse button pressed as long as they experienced the motion aftereffect, and released it as soon as the aftereffect dissipated. The duration of the motion aftereffect (i.e., the time between when the stimulus motion stopped and when the mouse button was finally released) was recorded as an index of the strength of adaptation to the prolonged exposure to the visual inducer’s motion, just as in several previous studies (e.g., Kim & Khuu, 2014; Palmisano et al., 2012). As in Experiment 1, there were two independent variables namely random-dot pattern oscillation and aperture oscillation.

### Results and Discussion

**Figure 5.**
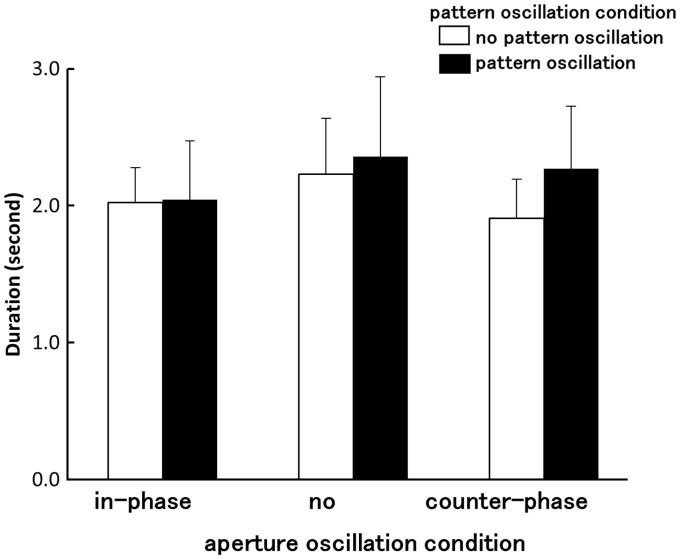
Average motion aftereffect durations measured under each stimulus condition (Experiment 3). Error bars indicate *SEMs*.

Figure 5 shows the averaged durations of the motion aftereffect obtained for each stimulus condition. A two-way rANOVA was used to analyze this motion aftereffect duration data. The main effects of pattern oscillation and aperture oscillation were not significant (pattern oscillation: *F*(1, 7) = 1.56, *p* = .25, pη^2 ^= .18; aperture oscillation: *F*(2, 14) = 1.30, *p* = .13, pη^2 ^= .25). Two-way interactions between pattern oscillation and aperture oscillation were also not significant (*F*(2, 14) = 2.17, *p* = .15, pη^2 ^= .23). Thus, the durations of the motion aftereffects generated by 30-second exposures to optic flow were not significantly altered by either pattern or aperture oscillation. Motion aftereffect durations were on average approximately 2 seconds long across all of the different conditions tested.

The main purpose of Experiment 3 was to determine whether differences in the degree of adaptation to the visual motion might account for the effects of stimulus oscillation on vection. Accordingly, we conducted a cross-experimental comparison of the vection (measured in Experiment 1) and the motion aftereffects (Experiment 3) produced by the various stimulus conditions. Aperture oscillation, or the relative oscillation between the pattern and the aperture, was shown to significantly alter vection onset latency. However, it did not modulate the motion aftereffect. Some previous studies have argued that adding visually simulated accelerations to the visual inducer might disrupt adaptation to optic flow, and that this reduced motion adaptation might be partially responsible for jitter or oscillation advantages for vection (e.g., Kim & Khuu, 2014). On the other hand, other studies have also failed to find significant correlations between vection strength and motion adaptation, similar to the current experiment (e.g., Palmisano et al., 2012). Thus, it appears that motion adaptation cannot fully explain vection increases caused by the addition of pattern and aperture oscillation in Experiment 1. Some consideration is therefore required when invoking the role of motion adaptation to account for vection.

## Experiment 4

In the psychophysical experiments reported in this article, we mainly discussed the effects of relative oscillation between the random-dot pattern and the aperture on vection. Observers always perceived that the aperture was located in front of the background random-dot pattern, even though no binocular disparity information was provided about the simulated depth separation (all of the components in the visual stimulus were presented coplanar on the screen). However, it was possible that manipulations of the relative oscillation between the aperture and the random-dot pattern altered the perceived depth represented by the display (via motion parallax), which in turn might have affected vection (e.g., Andersen & Braunstein, 1985). Experiment 4 tested this explanation by comparing the perceptions of display depth experienced for each stimulus used in Experiment 1.

### Methods

In this experiment, we measured the perceived depth separation between the aperture and the random-dot pattern (instead of the observer’s perceived self-motion). We used visual stimulus conditions that were identical to those of Experiment 1. Nine undergraduate students (4 men and 5 women, aged 19–21 years old) participated in this experiment (they had not taken part in Experiments 1–3). In the experimental trials, they estimated the perceived depth separation between the aperture and the random-dot pattern and verbally reported it. The standard stimulus for these depth estimates was again the no aperture and no pattern oscillation condition. The perceived separation in depth for this standard stimulus was assigned a value of “50” (the modulus for their subsequent magnitude estimates).

### Results and Discussion

**Figure 6.**
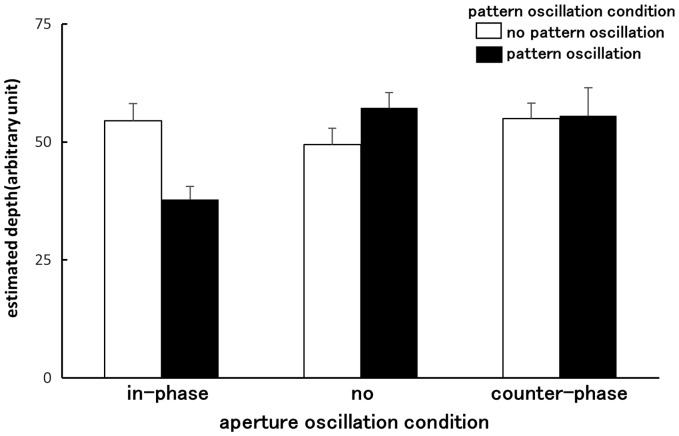
Average estimates of the depth separation between the aperture and the random-dot (Experiment 4). Error bars indicate *SEMs*.

Figure 6 shows the mean estimated depth separations between the aperture and the random-dot pattern for each stimulus condition. rANOVA revealed that the main effect of aperture oscillation was significant (*F*(2, 16) = 4.52, *p* = .028, pη^2 ^= .36). While the main effect of pattern oscillation was not significant (*F*(1, 8) = 1.06, *p* = .33, pη^2 ^= .12), the interaction between aperture oscillation and pattern oscillation was significant (*F*(2, 16) = 10.76, *p* = .001, pη^2 ^= .57). Multiple comparisons using Bonferroni correction (α = 0.05) indicated that perceived depth separation was significantly reduced (by ∼ 25%) in the in-phase aperture and random-dot pattern oscillation condition (compared with other conditions). No other differences in the depth perception were found.

In Experiment 1, we revealed that the in-phase aperture-and-pattern oscillation increased vection onset latency, indicating that self-motion perception was weakened in that condition. It is possible that the decline in vection observed for this condition might have been due to decreased depth perception. However, even in this condition, all of the observers still perceived the aperture to be in front of the random-dot pattern. Previous studies have also shown that it is the depth order among visual components (in terms of foreground–background relationships), not a depth separation between them, that is critical for self-motion perception (Nakamura, 2006b). Thus we would argue that it is unlikely variation in perceived depth accounted for the experience of vection in this case. It should also be noted that in the counter-phase aperture-and-pattern oscillation condition, there was no perceived depth enhancement, even though vection was facilitated in Experiment 1. Thus, at best, perceived depth can only partially account for the effect of relative oscillation on vection (decrement in the in-phase aperture oscillation condition).

## General Discussion

Our psychophysical experiments investigated the effects on vection of adding oscillation (directly and indirectly) to the visual inducer. A simulated nearby visual aperture was provided which could be set to oscillate independently of the primary vection inducer, that is, the moving background random-dot pattern. Experiment 1 revealed that horizontal aperture oscillation could alter the onset latency of vertical vection induced by the background flow—with the nature of this effect depending on the amount of relative oscillation between the random-dot pattern and the aperture. When the random-dot pattern only moved upward, horizontal aperture oscillation (in front of the pattern) reduced vection onsets—most likely due to the increased relative oscillation between these two visual components. When the pattern also oscillated, in-phase aperture oscillation appeared to weaken vection, whereas counter-phase aperture oscillation appeared to facilitate it (presumably by doubling the amount of relative oscillation compared with the no aperture oscillation condition). Although Experiment 1 found that horizontal aperture and pattern oscillation can improve vertical vection, Experiment 2 found that vertical pattern oscillation actually impaired vertical vection. However, unlike vertical pattern oscillation, vertical aperture oscillation did not appear to alter either the strength or the latency of vertical vection. Experiment 3 demonstrated that the effects of horizontal aperture and pattern oscillation on vertical vection could not be explained by reduced motion adaptation, as motion aftereffect durations did not appear to vary significantly as a function of the different conditions. Finally, Experiment 4 demonstrated that modulations in perceived display depth (arising due to differences in motion parallax) could not account for all of the effects of relative oscillation on vection.

### The Effects of Visual Aperture on Self-Motion Perception

In the psychophysical experiments reported here, we introduced a simulated aperture into the visual stimulus to manipulate relative motion against the visual inducer. This aperture limited the visual stimulation to a central circular area with a radius of 30° in visual angle. It is possible that the inclusion of this aperture weakened vection—as vection tends to increase with the area of visual motion stimulation (e.g., Nakamura & Shimojo, 1998). Consistent with this notion, average vection latencies in the present experiments were somewhat longer than might be expected in inducing conditions with a larger field-of-view. However, because this aperture was clearly seen to be in the foreground, it also strengthened participants’ perceptions that the moving random-dot pattern lay in the background (confirmed during debriefing). Previous studies have shown that compelling vection only occurs when the optic flow field is perceived to be in the background (e.g., Brandt et al., 1973; Nakamura & Shimojo, 1999; Ohmi, Howard, & Landolt, 1987). Indeed some researchers have hypothesized that vection is primarily induced by visual stimuli perceived to be in the background, and that visual factors which enhance this background perception boost self-motion perception (object-background hypothesis; Seno, Ito, & Sunaga, 2009; c.f., Kim & Tran, 2016). Thus, it is likely that the strengthening of the optic flow’s perceived background status (by the inclusion of the nearby aperture) partially compensated for any effects on vection of having a visual motion inducer that was of a limited size.

### The Effects of Relative Oscillation Between the Visual Pattern and the Aperture

The main purpose of this investigation was to examine the effect of relative oscillation between visual components (the random-dot flow field and an aperture) on vection. Experiment 1 revealed that vection latency decreased when a horizontally oscillating aperture was positioned in front of the self-motion inducing flow pattern. In that experiment, vection strength was best explained as a function of the relative motion between the random-dot pattern and the aperture; vection latency decreased in the counter-phase aperture oscillation condition (which should have doubled the speed of the relative motion compared with the no aperture oscillation condition), and increased during in-phase aperture oscillation (which should have diminished the relative motion between aperture and flow field). These results suggest that the oscillation advantage in vection is determined not only by the visual oscillation of the inducer itself but also by the relative oscillation between foreground and background visual elements (the aperture and the random-dot pattern, respectively, in this investigation). Several previous studies have revealed that vection is strongly affected by relative motion or motion contrast among visual components, which also influences other visual motion perceptions, namely Dunker illusion (e.g., Heckmann & Howard, 1991; Howard & Heckman, 1989; Howard & Howard, 1994). In debriefing sessions, our participants often reported that their perceptions of the horizontally oscillating random-dot patterns did depend on the motion of the aperture; perceived oscillation of the pattern was strengthened in the counter-phase aperture oscillation condition (where relative motion was doubled), and weakened when there was no relative motion in the in-phase aperture oscillation condition. We propose that the effect of aperture oscillation on vection in Experiment 1 can be explained by the strength of the perceived oscillatory motion modulated by the relative motion between the aperture and flow field.

It is also noteworthy that the estimated strength of vection was not significantly affected by aperture oscillation in the current study (only vection onset latency). This null finding might indicate that the effects of relative motion were moderately weak in the current experimental setup. The relative motion in our displays always existed only in restricted narrow regions at the edge of the simulated visual aperture (which was located 30° out from the center of the observer’s visual field). It is possible that such relative motion effects might be strengthened in future by introducing multiple small apertures covering the random-dot pattern, or using two overlapping random-dot patterns (similar to those used by Nakamura, 2013b; Nakamura & Shimojo, 1999, 2000). This would allow us to obtain much stronger relative motion compared with using only the one aperture in the present experiments. Through such investigation will we learn more about the effects of accelerating visual motion (jitter or oscillation) on vection, and self-motion perception more generally.

### The Possible Effects of Relative Motion Against the Fixation Spot

In the psychophysical experiments reported in this article, a static fixation spot was always presented on the center of the visual display. Thus, although we have concentrated on analyzing the effect of the relative oscillation between the aperture and the random-dot pattern, motions were actually relative to three different visual components, namely, the random-dot pattern, the aperture, and the fixation spot. In the condition with no pattern and no aperture oscillation, there was no relative oscillation with respect to any of these three visual components. In the in-phase pattern and aperture oscillation, while there was no relative oscillation between the pattern and the aperture, there was still relative oscillation of the pattern and aperture with respect to the static fixation spot. In Experiment 1, vection onset latency was significantly shorter in the “in-phase aperture oscillation” condition than in the “no pattern and no aperture oscillation” condition (see Figure 3). We propose that the relative oscillation between the pattern + aperture and the fixation spot decreased the onset of vection in the former condition (relative to the latter condition). In this situation, a single static visual object presented in the center of the visual field also appeared to serve as a useful reference for relative oscillation.

Palmisano et al. (2012) and also Nakamura (2013a) have previously reported that vection can be increased by making oscillatory eye movements (to follow a moving fixation target) when viewing a nonoscillating pattern of optic flow. These studies demonstrate that vection can be enhanced by retinal oscillation alone (even when there is no oscillation of the optic flow field; see also Kim & Palmisano, 2010). The current investigation also shows that a stationary (as opposed to moving) fixation target can facilitate vection by acting as a frame of reference for relative motion. Thus, these previous vection advantages (found when visually pursuing a moving fixation spot) may have been jointly generated by the retinal image acceleration (due to the eye movement) and the presence of a frame of reference for relative motion (due to the presence of the fixation spot). Future experiments should try to distinguish between the effects of retinal oscillation and relative motion on vection, by controlling or monitoring eye movement under various observational conditions either with or without explicit fixation target. This should provide exciting avenues to explore more parametrically the potential role of eye movements on relative motion, and in turn, self-motion perception.

## Supplementary Material

Supplementary material

## Supplementary Material

Supplementary material

## Supplementary Material

Supplementary material

## Supplementary Material

Supplementary material

## Notes

1 Consistent with this notion Palmisano and Kim (2009; Experiment 1B) also reported that viewpoint jitter produced the greatest vection improvements when participants either looked or fixated centrally (as opposed to peripherally) in the optic flow display.

2 Here we refer to “relative oscillation” as the horizontal oscillatory relative motion between the random-dot pattern and the aperture (i.e., velocity was sinusoidally modulated). This is differentiated from general relative motion because all of the displays tested had relative motion between the aperture and the constant upward motion of the random-dot pattern (even in the standard nonoscillating condition).
