# CORRIGENDUM to Relative Visual Oscillation Can Facilitate Visually Induced Self-Motion Perception

**DOI:** 10.1177/2041669516686459

**Published:** 2016-12-01

Shinji Nakamura, Stephen Palmisano, and Juno Kim (July-August 2016). Relative Visual Oscillation Can Facilitate Visually Induced Self-Motion Perception. *i-Perception*, *7*(4), 1–18. (DOI: 10.1177/2041669516661903).

Changes have been made to this article after its publication on August 5, 2016 issue of i-Perception. These corrections will be included in all subsequent versions of the article online.

On page 4, line 4, the following sentence should read as follows: As the fixation spot was always statically located at the center of the screen, this allowed us to determine the effects of relative motion on vection independently of the observer’s eye movements (and their flow-on consequences in terms of the retinal image motion).

In the **Author Biographies** section, the image for author Juno Kim was omitted and the biography should read as follows:

**Figure:**
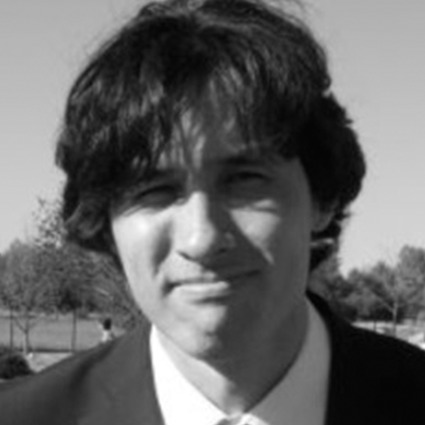

**Juno Kim** is a Senior Lecturer and Australian Research Council (ARC) Future Fellow appointed to the School of Optometry and Vision Science at the University of New South Wales. Dr. Kim has an extensive track record in research looking at the perceptual and physiological processes underlying visual experience. He is also committed to teaching and promoting the talents and achievements of early career researchers.

